# Acquired trigemino‐abducens synkinesis in a dog with immune‐mediated masticatory myositis

**DOI:** 10.1111/jsap.70079

**Published:** 2026-01-11

**Authors:** S. Garzón, T. Liatis

**Affiliations:** ^1^ Neurodiagnostico Veterinario Bogota Colombia; ^2^ Plakentia Veterinary Clinic Alimos Athens Greece

A 1‐year‐old male entire Rottweiler was presented with chronic (4 months) progressive jaw pain and trismus. Neurological examination revealed trismus, bilateral masticatory muscle swelling and myalgia. Within 2 weeks, there was progression to bilateral masticatory muscle atrophy and bilateral third eyelid protrusion while yawning despite steroid therapy (anti‐inflammatory dose). During attempted jaw opening, simultaneous retraction of the eye globe and protrusion of the third eyelid occurred bilaterally, suggestive of trigemino‐abducens synkinesis (Video 1). Haematology and serum biochemistry were within normal limits. Computed tomography of the skull showed diffuse swelling of the temporalis and masseter muscles, hyperattenuation and contrast enhancement, consistent with acute inflammatory myopathy. Serology for anti‐2M antibodies was positive at 1:4000, confirming immune‐mediated masticatory myositis. Prednisolone (2 mg/kg po q24h) was started. At a 14‐day follow‐up, marked clinical improvement was observed, with improved jaw mobility, partial reduction of synkinesis and persistent muscle atrophy. The synkinesis resolved completely 2 months post treatment, while at a 6‐month follow‐up, the masticatory muscles were normal. Trigemino‐abducens synkinesis is an abnormal involuntary movement involving activation of the abducens nerve (eye retraction) during voluntary trigeminal motor activity (mastication). Acquired trigemino‐abducens synkinesis has been rarely associated with inflammatory, traumatic or post‐surgical injury in humans. It can be a result of aberrant ephaptic transmission or maladaptive cross‐talk between trigeminal nerve motor fibres and their internuclear connections with abducens nerve, possibly exacerbated by the inflammatory process of the muscles in our case. Acquired trigemino‐abducens synkinesis can be a rare sign of immune‐mediated masticatory myositis in dogs.

**VIDEO 1:** A dog with immune‐mediated masticatory myositis and concurrent trigemino‐abducens synkinesis while chewing.

## Author contributions

**T. Liatis:** Conceptualization; writing – original draft; validation; writing – review and editing. **S. Garzón**: conceptualization; writing‐original draft; writing‐review and editing.

